# Picture quiz

**Published:** 2018-07-31

**Figure:**
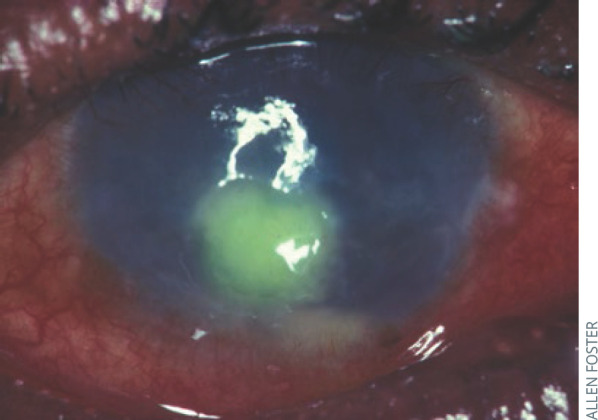


A 45 -year-old woman in a country with limited eye care services presents with a one-week history of a painful eye with loss of vision. There is no history of injury. After applying fluorescein to the conjunctival sac the appearance is as shown.

**Tick ALL that are TRUE**

**Question 1 What is the most likely diagnosis?**
  □ **a.** Conjunctivitis
  □ **b.** Iritis
  □ **c.** Herpes simplex viral keratitis
  □ **d.** Microbial keratitis
  □ **e.** Traumatic abrasion
**Question 2 What clinical signs are present?**
  □ **a.** Hyphaema
  □ **b.** Corneal ulceration
  □ **c.** Corneal vascularisation
  □ **d.** Hypopyon
  □ **e.** Trichiasis
**Question 3 What treatments might be useful in managing this condition??**
  □ **a.** Atropine eye drops
  □ **b.** Acyclovir eye ointment
  □ **c.** Epilation
  □ **d.** Prednisolone 0.5% eye drops
  □ **e.** Topical or sub-conjunctival antibiotics

## ANSWERS

1. d. There is evidence of corneal fluorescein staining, corneal infiltration and hypopyon consistent with a diagnosis of microbial keratitis.
2. b, c, d and e. There is no evidence of hyphaema; all other signs are present. Note the one broken off eyelash on the upper eyelid at about the 10 o'clock position of the cornea, which may be the cause of this corneal ulcer.
3. a, c, and e. This ulcer is unlikely to be due to herpes simplex virus as there is no characteristic branching pattern. Prednisolone is not indicated. Epilation is important to remove the inturned eyelash. Treatment with topical or sub-conjunctival antibiotics is essential.

